# HIV Drug Resistance Mutations and Subtype Profiles among Pregnant Women of Ho Chi Minh City, South Vietnam

**DOI:** 10.3390/v15102008

**Published:** 2023-09-27

**Authors:** Yulia V. Ostankova, Alexandr N. Shchemelev, Huynh Hoang Khanh Thu, Vladimir S. Davydenko, Diana E. Reingardt, Elena N. Serikova, Elena B. Zueva, Areg A. Totolian

**Affiliations:** 1 Saint Petersburg Pasteur Institute, 19710 St. Petersburg, Russia; shenna1@yandex.ru (Y.V.O.);; 2 HIV/AIDS Laboratory, Pasteur Institute, Ho Chi Minh City 700000, Vietnam; hhkthupasteur@gmail.com

**Keywords:** human immunodeficiency virus, HIV, HIV drug resistance, laboratory diagnostics, prevent mother-to-child transmission of HIV

## Abstract

According to the latest data released by UNAIDS, the global number of people living with HIV (PLHIV) in 2021 was 38.4 million, with 1.5 million new HIV infections. In different countries, a significant proportion of these cases occur in the adult fertile population aged 15–49 years. According to UNAIDS, Vietnam had a national HIV prevalence of 0.3% of the total population at the end of 2019, with approximately 230,000 PLHIV. The most effective way to prevent mother-to-child transmission of HIV is ART to reduce maternal viral load. HIV-infected pregnant women should undergo monthly monitoring, especially before the expected date of delivery. The aim of our work was to analyze subtypic structure and drug-resistant variants of HIV in pregnant women in Ho Chi Minh City. The study material was blood plasma samples from HIV-infected pregnant women: 31 women showed virological failure of ART, and 30 women had not previously received therapy. HIV-1 genotyping and mutation detection were performed based on analysis of the nucleotide sequences of the *pol* gene region. More than 98% of sequences genotyped as HIV-1 sub-subtype CRF01_AE. When assessing the occurrence of drug resistance mutations, genetic resistance to any drug was detected in 74.41% (95% CI: 62.71–85.54%) of patients. These included resistance mutations to protease inhibitors in 60.66% (95% CI: 47.31–72.93%) of patients, to NRTIs in 8.20% (95% CI: 2.72–18.10%), and to NNRTIs in 44.26% (95% CI: 31.55–57.52%). Mutations associated with NRTI (2) and NNRTI (8) resistance as well as PI mutations (12), including minor ones, were identified. The high prevalence of drug resistance mutations found in this study among pregnant women, both in therapeutically naive individuals and in patients with virological failure of ART, indicates that currently used regimens in Vietnam are insufficient to prevent vertical HIV infection.

## 1. Introduction

Currently, the human immunodeficiency virus 1 (HIV-1) problem has acquired global significance and the actual status of a pandemic. According to data as of the end of 2021, the virus has claimed more than 40 million human lives [1,2]. In most countries, the mortality rate is less than 10 per 100,000 people per year, and often much lower (<5 per 100,000), but mortality in Africa and Asia is still high. The highest mortality from HIV-1-related causes is seen among HIV-infected persons aged 15–49 years [3]. Approximately 4000 people are infected with HIV-1 every day. According to the latest data released by UNAIDS, the global number of people living with HIV (PLHIV) in 2021 was 38.4 million, with 1.5 million new HIV infections. In different countries, a significant proportion of these cases occur in the adult fertile population aged 15–49 years [3]. Despite the measures taken, and decreases in the number of new infections, the number of PLHIV is growing [3].

The introduction of highly active antiretroviral therapy (ART), the main goal of which is to suppress viral replication, significantly improves the prognosis for HIV-infected patients, reduces mortality and the number of HIV-related complications, and increases patient survival. ART is a combination of antiretroviral drugs (ARVs) that target various stages of HIV-1 replication. By acting on these stages, ART interrupts replication, thereby stopping reproduction of the virus in the cell. Proper use of ART has effectively turned an inescapably fatal disease into a treatable chronic condition by suppressing the viral load to undetectable levels and ensuring a sustained increase in CD4+ T-lymphocyte counts [4]. Alongside effective tools for proper prevention, HIV-infected individuals can live long and healthy lives when certain materials and procedures are implemented: modern ARVs, timely diagnosis and treatment of comorbidities, and proper overall patient care.

To achieve these goals, however, constant monitoring is required to assess HIV-1 prevalence and development of the epidemic situation, taking into account molecular genetic characteristics of regional viral strains. Despite significant improvement in HIV/AIDS rates after the introduction of ART, drug resistance of the virus remains a serious threat to the sustainable impact of ARVs worldwide [5]. The high rate of viral evolution, and its molecular genetic variability, lead to the accumulation of drug resistance (DR) mutations over time [6]. With HIV, simultaneous drug resistance (to several drug groups at once) is especially important since this significantly reduces therapeutic options. In fact, treatment may fail in 16–27% of non-ART patients and in 50–70% of previously treated patients [7].

Various estimates indicate that the incidence of HIV in the Socialist Republic of Vietnam (Vietnam) peaked in early 2000 but has declined significantly since then. According to UNAIDS, Vietnam had a national HIV prevalence of 0.3% of the total population at the end of 2019, with approximately 230,000 PLHIV [8]. The HIV-1 epidemic in Vietnam is still concentrated in three key populations: men who have sex with men (MSM) (12.2% prevalence), persons who inject drugs (PWID) (14.0% prevalence), and female sex workers (FSW) (3.6% prevalence) [8].

Since 2010, however, the number of new HIV infections in Vietnam has fallen by 65% [2,3,8]. Compared to 2015 levels, the number of new infections through blood (i.e., needle sharing) has decreased by 57%, and the number of new infections through sexual contact has decreased by 34.7% [3,8]. However, the fading of the epidemic remains unstable and insufficient. For example, only 65% of MSM are estimated to be aware of their status, and 23% of MSM living with HIV reported last receiving ART in 2018 [8]. In addition, the number of newly diagnosed HIV infections continues to rise in some areas, in particular in the Vietnam’s largest cities (Hanoi and Ho Chi Minh City) as well as in remote mountainous provinces (Dien Ben, Son La, Nghe An, and Thanh Hoa) [9].

According to the Maternal and Child Health Department of the Ministry of Health, there are about 2 million pregnant women in Vietnam annually, and HIV infection among pregnant women is estimated at 0.19%, representing more than 3800 pregnant women infected with HIV. The most effective way to prevent mother-to-child transmission (PMTCT) of HIV is ART to reduce maternal viral load. HIV-infected pregnant women should undergo monthly monitoring, especially before the expected date of delivery. The Ministry of Health of Vietnam has developed a decentralized PMTCT model, based on timely detection of HIV and early initiation of antiretroviral therapy (ART) for HIV-positive pregnant women. PMTCT is implemented in regions with high HIV population and includes additional measures such as early provision of ART to children. Medical staff are also trained to implement PMTCT and create proper conditions to manage social and psychological problems associated with HIV/AIDS. In Vietnam, the following antiretroviral drugs are used to prevent mother-to-child transmission of HIV: nevirapine, which is widely used for HIV prevention from mother to child, and is usually prescribed to HIV-positive pregnant women during subsequent trimesters of pregnancy and for several weeks after childbirth; zidovudine, another antiretroviral drug used to prevent HIV infection in newborns in Vietnam; and lopinavir/ritonavir, which is sometimes used to prevent HIV in newborns in Vietnam, especially if the mother received antiviral therapy during pregnancy.

By the end of 2017, women detected as HIV-positive later than the 24th week of pregnancy were advised to take a regimen of TDF+3TC+RAL (raltegravir). Since 2020, a fixed-dose combination of TDF+3TC+DTG (dolutegravir) has been recommended as the preferred regimen for all pregnant women living with HIV. In practice, however, EFV was continually used due to the shortage of regimens containing DTG or RAL in Vietnam. For infant prophylaxis, a single dose of nevirapine (sdNVP) within 6 to 12 weeks of birth was recommended for HIV-exposed infants. Since 2018, sdNVP for 6 weeks postpartum has been applied for cases with a low risk of vertical transmission, and a combination of AZT (zidovudine) plus NVP has been used for 6 weeks among infants with a high risk of transmission or 12 weeks if high-risk and being breastfed [10,11,12].

There is practically no information in the literature about cases of the inefficiency of PMTCT in Vietnam; however, according to some reports, the accumulation of major drug resistance mutations was found in 43.8% of infants, and most of them were resistant to non-nucleoside reverse transcriptase inhibitors (NNRTIs) (37.7%). High-level resistance to nevirapine was present in 40% of cases [13].

The risk factors associated with NNRTI resistance were antiretroviral prophylaxis (aOR: 3.68, 95% CI: 1.83–7.45) and breastfeeding history (aOR: 2.16, 95% CI: 1.03–4.53).

The aim of our work was to analyze subtypic structure and drug-resistant variants of HIV in pregnant women in Ho Chi Minh City.

## 2. Materials and Methods

This study was approved by the Ethics Committee of the Saint Petersburg Pasteur Institute.

The study material was blood plasma samples collected in 2021 from 61 pregnant women in need of PMTCT in Ho Chi Minh City with a viral load above 500 copies per ml of blood plasma. The blood plasma after taking the material from women was sampled, and then sent to the Pasteur Institute in Ho Chi Minh City, where it was frozen at −70 °C. After the sampling was completed, the frozen plasma was transported to St. Petersburg, where molecular biological studies were carried out. In the surveyed group, 31 women showed virological failure of ART, and 30 women had not previously received therapy. All samples were transferred anonymously.

Molecular genetic studies were carried out at the Pasteur Institute in St. Petersburg. Quantitative analysis of HIV RNA was carried out with a commercial kit, AmpliSens^®^ HIV-Monitor-FRT (Central Research Institute of Epidemiology, Moscow, Russia) with a sensitivity threshold of 500 copies/mL. For samples with a detectable viral load, reverse transcription and PCR were performed, followed by Sanger sequencing using the AmpliSens HIVResist-Seq (Central Research Institute of Epidemiology, Moscow, Russia) commercial kit [14]. HIV-1 genotyping was performed based on analysis of the nucleotide sequences of the 1285 nt *pol* gene region encoding the protease (PR) and part of the reverse transcriptase (RT) in the 2085–3369 nt region. The coordinates given are for HIV HXB2 in the GenBank database (K03455.1). Sequencing reaction products were analyzed using an ABI Prism 3500 genetic analyzer (Applied Biosystems, Waltham, MA, USA).

Nucleotide sequences were aligned using the MEGA 7.0 program using the ClustalW algorithm [15]. To construct phylogenetic trees and subsequent phylogenetic analysis, the neighbor-joining algorithm was used, which makes it possible to optimize trees in accordance with the balanced minimum evolution criterion. When assessing the reliability of phylogenetic relationships, we used multiple generation of samples using the bootstrap method for 1000 independent constructions of each phylogenetic tree. Genotyping of the studied strains was carried out in parallel using the REGA HIV-1 Subtyping Tool 3.0 program [16] based on analysis of phylogenetic relationships with reference sequences from the GenBank international database. Analysis of HIV-1 genetic sequences for the presence of drug resistance mutations was performed using the Stanford database [17]. Mutation profiles were analyzed by constructing line diagrams using Linear Diagram Generator software [18].

Statistical data processing was carried out using the MS Excel 2016 and Prizm 5.0 (GraphPad Software, Inc., San Diego, CA, USA) software packages. The “exact” Clopper–Pearson interval was used to estimate statistical uncertainty. Results are represented as a proportion indicating 95% confidence interval (95% CI). The Fisher exact test or Yates-corrected chi-squared test was used to evaluate the statistical significance of numeric data obtained during paired comparison depending on sample characteristics. A probability value of *p* < 0.05 was taken as the statistical threshold of significance.

## 3. Results

**Figure 1:**
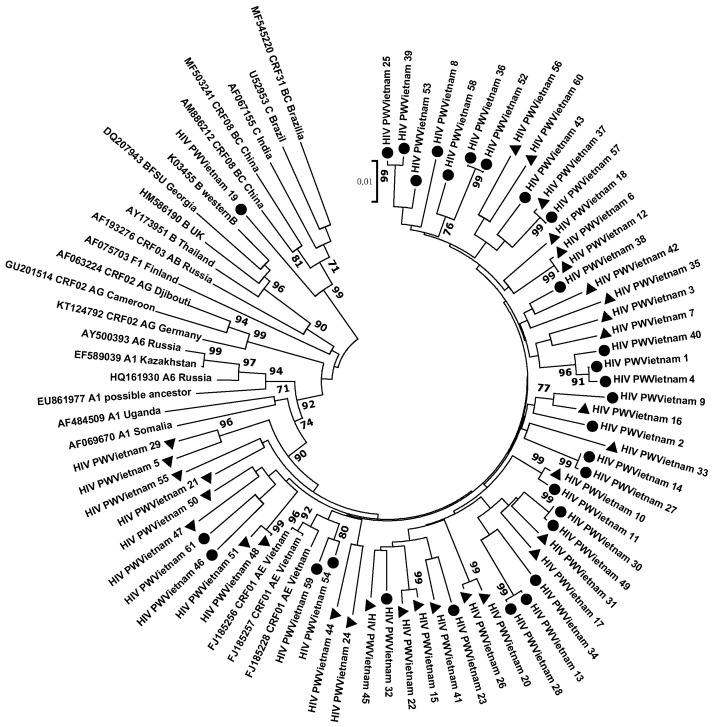
Phylogenetic analysis of viral nucleotide sequences (HIV *pol* gene fragment) from HIV-infected pregnant women in Vietnam relative to GenBank reference sequences. Reference sequences are designated by GenBank codes indicating the sample genotype. Strains studied in this work are marked as follows: triangles—samples from patients with virologically ineffective ART; circles—samples from patients who had not previously received therapy. Bootstrap values ≥70% are given.

The nucleotide sequences of 61 HIV-1 samples were obtained and deposited in GenBank (numbers OQ215322–OQ215382). The subtype was determined for all samples (Table 1). For this, data obtained by genotyping using the REGA HIV Subtyping Tool 3.0, the jumping profile Hidden Markov Model (jpHMM), and the results of a phylogenetic study (Figure 1) were used.

When assessing the occurrence of drug resistance mutations, genetic resistance to any drug was detected in 74.41% (95% CI: 62.71–85.54%) of patients. These included resistance mutations to protease inhibitors in 60.66% (95% CI: 47.31–72.93%) of patients, to NRTIs in 8.20% (95% CI: 2.72–18.10%), and to NNRTIs in 44.26% (95% CI: 31.55–57.52%). Mutations associated with NRTI (2) and NNRTI (8) resistance as well as PI mutations (12), including minor ones, were identified.

**Figure 2:**
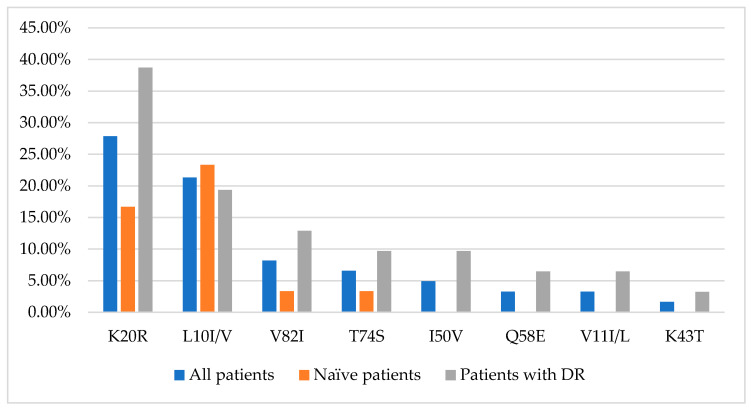
Most common HIV drug resistance mutations to PIs in HIV-infected pregnant women in the Socialist Republic of Vietnam.

**Figure 3:**
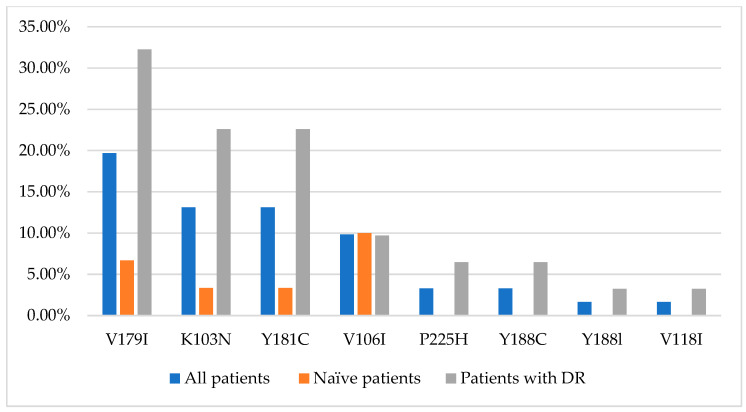
Most common HIV drug resistance mutations to NNRTIs in HIV-infected pregnant women in the Socialist Republic of Vietnam.

NRTI mutations were present only in women with virological failure of ART. PI and NNRTI mutations were also detected in therapeutically naive patients. The prevalence values of HIV drug resistance mutations to PIs and NNRTIs in the examined group are shown in Figure 2 and Figure 3, respectively.

The occurrence of mutations in the studied subgroups was not the same (Table 2).

**Figure 4:**
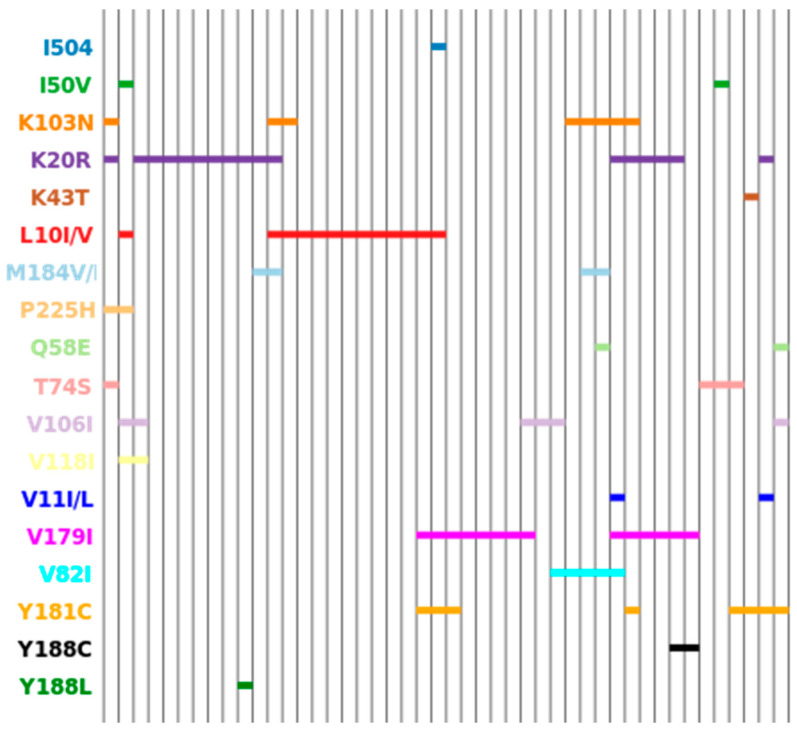
Overall analysis of multiple mutation profiles by line diagram. Mutation profiles are distributed along the horizontal axis.

**Figure 5:**
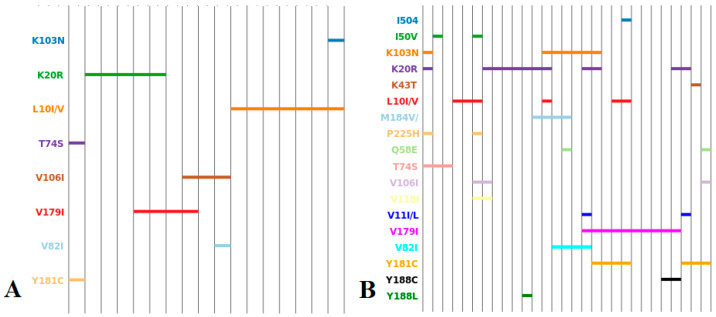
Analysis of multiple mutation profiles by line diagram. Therapeutically naive individuals (**A**) and individuals with failure ART (**B**). Mutation profiles are distributed along the horizontal axis.

By analyzing multiple mutation profiles by constructing line diagrams, it is possible to trace emerging stable patterns of drug resistance mutations (Figure 4 and Figure 5).

The prevalence of any mutation in women with virological failure of ART was significantly higher (93.55%, 95% CI: 78.58–99.21%) than in women who had not previously received treatment (56.67%, 95% CI: 37.43–74.54%), with the values χ^2^ = 9.283, *p* = 0.0023, df = 1, and RR = 4.728 (CI: 1.277–17.513). In addition, polymorphic mutations were seen in both examined subgroups, while non-polymorphic drug resistance mutations were noted predominantly in individuals with ineffective ART (67.74%, 95% CI: 48.63–83.32%). The incidence of DR mutations in therapeutically naive individuals was also extremely high for this group, 10% (95% CI: 2.11–26.53%). However, the relative risk of developing DR mutations in individuals with virological failure of ART was significantly higher than in those who had not received treatment, with the values χ^2^ = 18.949, *p* < 0.0001, df = 1, and RR = 3.238 (CI: 1.866–5.616).

## 4. Discussion

The results obtained have a number of limitations and, undoubtedly, require further refinement. The main limitation is the small size of the studied sample of patients. Some difficulty in interpreting the data is created by the poor availability of some information from the anamnesis, including the complete anonymity of the samples transmitted to us for work. Nevertheless, based on the obtained results, it is possible to make a number of assumptions, conducting a comparative analysis with other information presented in the literature.

It is known that CRF01_AE was the first-described circulating recombinant form of HIV-1. Moreover, although the genetic variant was first identified in Thailand, its origin from African countries has been determined [19]. However, the highest prevalence of CRF01_AE has been shown in Southeast Asian countries [20]. Our results (98.36% CRF01_AE, 1.64% C) differ slightly from the distribution of HIV genetic variants shown earlier in Vietnam. For example, in central Vietnam among HIV-infected individuals from different risk groups, CRF01_AE prevailed (97.5%) compared to genotype B (2.5%); despite an absence of therapy in these patients, 32.5% had at least one drug resistance mutation [21]. We showed a similar structure of HIV genotypes when examining patients with virological failure of ART from Ho Chi Minh City: the circulating recombinant form CRF01_AE (92.2%) prevailed compared to genotype B (5.3%), and CRF08_BC was detected in one patient (2.6%) [22]. In the present study, we did not detect HIV genotypes B or CRF08_BC, which may be due to the limited sample size. Thus, the predominance of this HIV genetic variant in the examined group does not contradict the previously obtained data in general [23]. The predominant prevalence of HIV CRF01_AE is also shown in neighboring countries of Vietnam [24]. For example, in Cambodia, which has one of the highest HIV prevalence among pregnant women in the Asia–Pacific Region, about 94% of patients with newly diagnosed HIV infection were infected with the CRF01_AE genovariant [25]. In Laos, 91.8% of HIV cases were CRF01_AE, and 8.2% of the strains were identified as a new circulating recombinant form of CRF97_01B; moreover, according to phylogeographic analysis, HIV CRF01_AE in Laos is the result of repeated importation of the virus from Thailand [26]. Generally, in Cambodia, Thailand, Vietnam, and China, the proportion of HIV-1 infections due to recombinants is more than 95% of cases [27]. At the same time, according to a retrospective study, in general, in the countries of the Asia–Pacific Region, HIV genotype C (49%) prevailed compared to genotype A1 (22%) and CRF01_AE (17%) [28]. These differences appear to be related to the characteristics of the patient groups in each study.

Our study, already mentioned above, showed a high prevalence of drug resistance mutations among individuals with virological failure of ART (76.2%), but strains with DR mutations to all three ARV groups simultaneously were not detected [22]. In the present study, three cases with mutations to NRTIs and NNRTIs were identified among people with failure ART with a mutation in the protease region. However, in all these cases, mutations in the protease region were polymorphic amino acid substitutions that increase the ability of the virus to replicate. We previously reported that the number of naturally occurring polymorphs in this region among ART users ranged from 17 to 35 mutations and also depended on the number of ART regimens. It was shown that ≥23 polymorphic variants in patients with two or three ART regimens are more common than in patients with one regimen, with the values χ^2^ = 3.149, *p* = 0.035, df = 1, and Pearson’s normalized contingency coefficient C′ = 0.459. This indicates a relatively strong relationship between a higher number of ART regimens and the number of polymorphic variants in the analyzed *pol* gene fragment [22].

In the present study, polymorphic mutations were found to be fewer, most likely due to the lack of ART or the single therapy regimen that women received. The results obtained indirectly confirm the assumption that a large number of ART regimens contribute not only to the development of drug resistance mutations, but also to an increase in the frequency of viral polymorphic variants. As is known, HIV genotypes have their own molecular genetic features which affect, among other things, sensitivity to ART. In Vietnam, available three-drug first-line ART regimens meet WHO standards: two NRTIs (stavudine (d4T)/lamivudine (3TC) or zidovudine (AZT)/lamivudine) together with one NNRTI (nevirapine (NVP) or efavirenz (EFV)). It is known that the effectiveness of ART is affected by the viral genotype, as well as HIV mutations present before the start of treatment [29]. For the CRF01_AE genotype, which is the most common in Vietnam (according to both the literature data and our results), the following mutations are among the most commonly described: L33F, M46I, and M46L (associated with DR to protease inhibitors); M41L, A62V, M184V, and T215Y (associated with DR to NRTI); and K103N, V108I, and G190A (associated with DR to NNRTI) [30,31].

In the examined strains, however, the NRTI mutations M41L, A62V, and T215Y were not found; the most common was M184V/I at 6.56% (95% CI: 1.82–15.95%). Among NNRTI mutations in our isolates, K103N and Y181C prevailed with 13.11% each (95% CI: 5.84–24.22%), while the V108I and G190A substitutions were not detected. Mutations L33F, M46I, and M46L were not found among PI mutations in the studied sequences. The most common mutations in our study group were polymorphic substitutions in the protease region, K20R (27.87%, CI: 17.15–40.83%) and I10I/V (21.31%, CI: 11.86–33.68%), which increase the replication of viruses with other PI-resistance mutations. Mutations of drug resistance to the protease inhibitor darunavir have been identified. The V11I/L mutations are relatively additional mutations selected from individuals receiving DRV. The V11L is a PI-selective mutation associated with reduced susceptibility to DRV in vitro when it occurs in combination with other PI-resistance mutations. In three women (4.92%), we identified the I50V mutation, selected by FPV, LPV, DRV, and reduced susceptibility to LPV and DRV. Thus, prevailing mutations of pharmacological resistance among the studied isolates were to lamivudine and emtricitabine (M184V); to delavirdine, nevirapine, and efavirenz (K103N); and to nevirapine, efavirenz, etravirine, and rilpivirine (Y181C). This high prevalence of drug resistance mutations for the most commonly used drugs is not surprising but is a concern as it limits their use in downstream regimens, especially given resistance to newer-generation drugs such as etravirine [32].

It should be noted that some polymorphic variants of the virus can also affect resistance to therapy, as well as the replication activity of the virus [33]. In the examined group, mutations were found in the V82I protease region (a highly polymorphic mutation that is not selected by the PI). The V82L mutation results in moderate levels of resistance to NFV and TPV, as well as low levels of resistance to ATV, FPV, and SQV [34]. A mutation at position 82 usually occurs simultaneously with a substitution at position 88. However, in our study, none of the isolates with the V82L mutation showed an amino acid substitution at position 88. At the same time, in all these isolates, the L89M polymorphism was detected, which contributes to resistance to a number of protease inhibitor drugs [35]. An amino acid substitution at this position of L89T occurs in 8.4% of non-B subtype sequences but only in 0.4% of B subtype sequences [36]. The absence of a substitution at position 88 simultaneously with polymorphism 82, but the presence of a therapeutically significant substitution at position 89 may be associated with the features of CRF01_AE, which needs further research. Note the T74S polymorphism found in some patients (is an additional mutation selected by PI that is polymorphic in most subtypes). Interestingly, the T74S amino acid substitution has not been described as being associated with drug resistance, but a mutation at this position in T74P is known to cause resistance to darunavir/ritonavir and tipranavir/ritonavir [35]. At the same time, although the T74S polymorphism itself does not affect the resistance of the virus, its presence in strains with multiple resistance restores sensitivity to indinavir, ritonavir, and partially to lopinavir [37]. The Q58E mutation has been identified. The Q58E mutation—a minimum polymorphic additional mutation selected by each of the PIs except DRV—in combination with other PI resistance mutations, may contribute to low levels of ATV resistance. Lopinavir/ritonavir is recommended as second-line therapy, for which one of the major Q58E mutations has little effect. Due to the lack of influence of Q58E on the sensitivity of strains to LPV/r, this mutation can serve as one of the markers for the transmission of specific quasi-species [38]. In addition, the high occurrence of polymorphic substitutions may indicate an active mutational process, as a result of which the active use of PI when using first- or second-line regimens that include ineffective drugs can potentially lead to the development of cross-resistance to modern drugs used in the second and third therapy regimens, such as darunavir [39]. This is due to the fact that the development of resistance to IP is cumulative, representing a gradual accumulation of amino acid substitutions [40].

The most common drug resistance mutations in our study cohort are consistent with other investigators’ evaluations of DR mutations in this geographic region [41]. So, in a retrospective study evaluating HIV testing results in individuals with virologic failure in countries of the Asia–Pacific Region, a high prevalence of drug resistance mutations was shown: 80% had at least one NNRTI mutation, 63% to NRTI and 35% to PI. The most common mutations to NNRTI were K103N (25%) and Y181C (25%); to NRTI-M184V (55%), M41L (35%) and D67N (28%); and to PI-I50L (10%), Q58E (10%), N88S (10%), and L90M (10%) [23].

Vertical transmission of HIV is primarily associated with infection during the last weeks of pregnancy and in the perinatal period. In this case, a significant role is played not only by high maternal HIV viral loads but also by drug resistance mutations [42]. Our results indicate the need to change ART in a significant number of pregnant women. Our study shows a high prevalence of DR mutations both in women with virological failure of ART and, to a lesser extent, in women who have not previously received treatment. Thus, due to time constraints, it is necessary to detect DR mutations before initiation of therapy in order to avoid prescribing drugs to which the virus is resistant. However, the high cost of testing may be a barrier to screening for DR mutations in Vietnam. In this case, attention should be paid to the possibility of using drugs for the prevention of infection in children that were not received by their mothers and which cannot have cross-drug resistance. Foremost, integrase strand transfer inhibitors (INSTIs), which have a high genetic barrier to drug resistance, should be considered [43]. INSTIs are recommended as the preferred ART components for initiation of therapy due to their efficacy, safety, and relative ease of use [44].

## 5. Conclusions

The high prevalence of drug resistance mutations found in this study among pregnant women, both in treatment-naive individuals and in patients with virological failure of ART, may indicate that currently used regimens in Vietnam are insufficient to prevent vertical transmission of HIV. Our results indirectly confirm the need to include integrase inhibitors in the first ART regimen, especially in pregnant women, parturient women, and newborns. However, it is important to note the main limitation of this study—a small sample size. Therefore, it is necessary to conduct a similar but larger-scale study. Nevertheless, the analysis of the prevalence of drug resistance mutations among pregnant women remains a necessary and mandatory step in HIV monitoring.

## Figures and Tables

**Table 1:** Distribution of strains by HIV-1 subtype.

Subtype	Number of Strains	Sample Share, %	95% CI, %	
			−	+
HIV-1 CRF01_AE	60	98.36	91.20	99.96
HIV-1 subtype C	1	1.64	0.04	8.80

**Table 2:** Drug resistance mutations in patients with and without treatment experience.

Mutation	Number of Naive Patients (N = 30)	Part of Naive Patients, %	Number of Patients with ART Failure (N = 31)	Part of Patients with ART Failure, %
K20R	5	16.67%	12	38.71%
L10I/V	7	23.33%	6	19.35%
V82I	1	3.33%	4	12.90%
T74S	1	3.33%	3	9.68%
I50V			3	9.68%
Q58E			2	6.45%
V11I/L			2	6.45%
K43T			1	3.23%
M184V/I			4	12.90%
V118I			1	3.23%
V179I	2	6.67%	10	32.26%
K103N	1	3.33%	7	22.58%
Y181C	1	3.33%	7	22.58%
V106I	3	10.00%	3	9.68%
P225H			2	6.45%
Y188C			2	6.45%
Y188L			1	3.23%
V118I			1	3.23%

## Data Availability

Data available on request from the authors.
